# Developing a dashboard to help measure and achieve the triple aim: a population-based cohort study

**DOI:** 10.1186/1472-6963-14-363

**Published:** 2014-08-30

**Authors:** Hsien-Yeang Seow, Lyn M Sibley

**Affiliations:** Cancer Care Ontario Research Chair in Health Services Research, Department of Oncology, Centre for Health Economics and Policy Analysis, McMaster University, 699 Concession St, 4th Fl, Rm 4-229, Hamilton, L8V 5C2 Ontario Canada; Primary Care and Population Health Division, Institute of Clinical Evaluative Sciences, Bldg G1-06, 2075 Bayview Avenue, Toronto, Ontario M4N 3M5 Canada

**Keywords:** Healthcare quality indicators, Quality improvement, Healthcare quality assurance, Risk adjustment, Canada

## Abstract

**Background:**

Health system planners aim to pursue the three goals of Triple Aim: 1) reduce health care costs; 2) improve population health; and 3) improve the care experience. Moreover, they also need measures that can reliably predict future health care needs in order to manage effectively the health system performance. Yet few measures exist to assess Triple Aim and predict future needs at a health system level. The purpose of this study is to explore the novel application of a case-mix adjustment method in order to measure and help improve the Triple Aim of health system performance.

**Methods:**

We applied a case-mix adjustment method to a population-based analysis to assess its usefulness as a measure of health system performance and Triple Aim. The study design was a retrospective, cohort study of adults from Ontario, Canada using administrative databases: individuals were assigned a predicted illness burden score using a case-mix adjustment system from diagnoses and health utilization data in 2008, and then followed forward to assess the actual health care utilization and costs in the following year (2009). We applied the Johns Hopkins Adjusted Clinical Group (ACG) Case-Mix System to categorize individuals into 60 levels of healthcare need, called ACGs. The outcomes were: 1) Number of individuals per ACG; 2) Total system costs per ACG; and 3) Mean cost per person per ACG, which together formed a health system “dashboard”.

**Results:**

We identified 11.4 million adults. 16.1% were aged 65 or older, 3.2 million (28%) did not use health care services that year, and 45,000 (0.4%) were in the highest acuity ACG category using 12 times more than an average adult. The sickest 1%, 5% and 15% of the population use about 10%, 30% and 50% of total health system costs respectively. The dashboard measures 2 dimensions of Triple Aim: 1) reduced costs: when total system costs per ACG or when average costs per person is reduced; and 2) improved population health: when more people move into healthier rather than sicker ACGs. It can help to achieve the third aim, improved care experience, when ACG utilization predictions are reported to providers to proactively develop care plans.

**Conclusions:**

The dashboard, developed via case-mix methods, measures 2 of the Triple Aim goals and can help health system planners better manage their health delivery systems.

## Background

Health system planners and policymakers are responsible for managing health system performance, and thus review quality indicators as well as assessments of the current illness burden in the population so as to help predict the future need for health care services. They need to use existing data to help them first measure health system performance, and second improve the quality of the health system, such as where to implement particular quality improvement activities. The Institute of Healthcare Improvement, an organization that is a leader of quality improvement and performance measurement at the health system level [1–4], has espoused the seminal concept that high performing health systems ought to pursue the Triple Aim: to 1) reduce health care costs; 2) improve population health; and 3) improve the care experience [5]. Within the U.K and U.S. especially, the Triple Aim has grown in popularity because new health care reforms, such as the NHS Outcomes Framework and the Affordable Care Act respectively, have broad goals of improving health system performance, such as improving quality, equity and access, but also include the three goals of the Triple Aim.

Within the past decade, many health system performance frameworks have been developed [6–23], comprised of various quality indicators, some of which relate to the Triple Aim goals. For example, quality indicators related to health system costs include health expenditures per capita [19], 30 day acute care readmissions [21], and preventable hospitalizations [22]. Population health has been assessed through indicators such as life expectancy, disability-adjusted life year [19], obesity rates in children, and the percentage of adults who smoke [22]. Care experience has been demonstrated by indicators such as the rate of adverse events, timely access to primary care, and wait times for surgery. On the one hand, the existing quality indicators have been useful to improve quality within particular populations because many of them are often disease-focused, setting-specific, or based on one episode of care. They have a well-defined denominator and thus a particular process of care or group of providers that can be held accountable to improve the rates. On the other hand, these measures are limited in other ways. They typically do not capture performance of the broader health care system, including care across different providers and settings throughout the continuum of care. As such current measures especially do not serve well patients with multiple comorbidities who represent the most expensive and medically-complex users of the system [24–26]. Thus it remains a challenge to measure the broader system performance, with respect to the Triple Aim.

Not only do planners want to measure Triple Aim and health system performance effectively, but want to do so in efficient and simple ways that can aid quality improvement. Health system planners and policymakers often use visual dashboards to integrate and compile important quality indicators and other key performance indicators into one place. An effective dashboard can help them to easily access and analyze important trends from the indicators, supporting timely decision-making and quality improvement. While other dashboards of health system performance exist, to our knowledge, no dashboards exist that measure the Triple Aim. Moreover, little research has explored how health system planners might use such as dashboard to aid quality improvement activities and help achieve the Triple Aim. The purpose of this study is to develop a visual dashboard, based on a case-mix adjustment method, and explore its applicability to measure and help improve health system performance, with respect to the Triple Aim.

## Methods

We conducted a retrospective, population-based, cohort study of adults from Ontario, Canada using multiple administrative databases. To develop the dashboard generally, individuals were assigned a predicted illness burden score using a case-mix adjustment system from diagnoses and health utilization data occurring in fiscal year 2007–08, and then followed forward to assess the actual health care utilization and costs in the following year (fiscal year 2008–09).

Specifically, we identified a cohort of unique Ontarians with a valid Ontario Health Insurance Plan number, the universal provincial health insurance plan, who were alive on April 1, 2008 and deterministically linked with the other administrative databases [27–32]. We excluded children aged younger than 18 to simplify the analysis. Using the previous fiscal year’s administrative data (FY2007-08), we applied the Johns Hopkins Adjusted Clinical Group (ACG) Case-Mix System (version 7) in this cohort to categorize individuals into mutually exclusive levels of healthcare need, called ACGs. Then using the subsequent fiscal year’s administrative data (FY2008-09), we determined the actual health care utilization and costs for each individual during that year. In doing so, this methodology aims to demonstrate the value of the dashboard to predict future costs and utilization, based on previous utilization and illness burden.

### Setting

In Ontario, Canada, because of the mainly single-payer nature of the health care financing system, the government collects a large amount of administrative health care data that can be used for research purposes. The provincial Ministry of Health and Long-Term Care provides funding for all Ontarians for hospital and physician services free at the point of care, with other services heavily subsidized. As such, Ontario’s Ministry of Health, in some ways, can be viewed as a large health insurer with approximately 13 M enrollees, and an annual budget of approximately $42B.

### Data Sources

The Registered Persons Database was the source for valid health insurance number, age (at start of FY), and sex. The Discharge Abstract Database, maintained by the Canadian Institute of Health Information, contained data for all hospital in-patient admissions and length of stay. The National Ambulatory Care Reporting System contained data for all emergency department (ED) and hospital and clinic outpatient visits. Finally, the Ontario Health Insurance Plan Claims database contained information on physician claims and ancillary services (e.g. laboratory work, blood tests).

### The ACG case-mix system

The Johns Hopkins ACG system is a diagnosis-based, case-mix method that uses administrative data to predict a population’s current and future healthcare need, healthcare use, and costs in one to two years. It captures the clinical complexity of multiple comorbidities without being disease-specific. ACGs have traditionally been used for risk-adjusted reimbursement and practitioner profiling in the US [33–37], Canada, and internationally [29, 38–44]. The method uses each person’s inpatient and outpatient diagnosis codes, age, and sex to assign individuals into 32 Ambulatory Diagnosis Groups (ADGs) — the building blocks of the ACGs. ADGs are unique in that they do not group by disease, but instead based on clinical similarities in need and expected resource use. Because people can have multiple chronic conditions, ADGs are not mutually exclusive. For example, ADG1 represents “time limited: minor conditions” (e.g. bell’s palsy or diaper rash), whereas ADG24 represents “injuries/adverse effects: major” (e.g. lower limb fractures or intracranial injury). Second, the methodology groups ADGs into 60 commonly occurring combinations, which are mutually exclusive ACG groups. Examples of ACG groups range from relatively small needs, such as “acute minor” (e.g. ear infection or cold), to the most expensive, “10+ ADG combinations, 4+ major ADGs” (e.g. multiple, unstable, chronic conditions with acute complications) [43].

### The dashboard

At the individual-level, based on FY2007-08 administrative data, every adult was categorized into an ACG, and their subsequent year’s health care utilization and costs from FY2008-09 were determined. Our results were aggregated into sub-populations of ACG categories, and the all 60 ACGs collectively represent all adults in the province. Specifically, we analyzed and presented our data to create 3 figures, which we refer to as a health system “dashboard”. The dashboard’s 3 figures describe the:

1. Number of individuals in each ACG;
2. Total system costs for each ACG; and
3. Mean cost per person for each ACG.

We applied a costing methodology that has been validated previously that includes health utilization costs of only hospitalizations, ED visits, physician claims and ancillary services claims that were publicly financed by the Ontario government [45–47]. Thus, the dashboard represents the past illness burden, utilization, health system costs, as well as the predicted health care use of the entire population profile or of any particular ACG category. We created a dashboard for the overall province.

Besides ACG categories, health care need was also quantified using ACG utilization weights, calibrated to Ontario data [43]. A utilization weight for each ACG represents the multiplier of expected resource use relative to the population’s mean expenditures. By definition, the average ACG weight was 1.0 for the entire population. Thus, an ACG weight of 2.0 represents a two-fold increase in expected healthcare utilization in the subsequent year. Note the dashboard is sorted from lowest to highest ACG weight, which corresponds to lowest to highest predicted healthcare need. Furthermore, we sorted our ACG categories into resource utilization quintiles based on the ACG weights. Higher quintiles were associated with higher expected utilization levels. All individuals in a given quintile were expected to use approximately the same amount of care. Non-users were excluded from the quintiles and assigned a zero value. This study was reviewed by the ethics committee of the Sunnybrook Health Science Centre and deemed exempt research, as it uses de-identified secondary data analysis.

## Results

**Figure 1:**
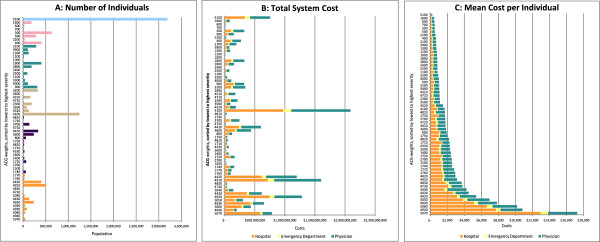
**Using the dashboard to help measure the Triple Aim.**

Our provincial analysis of Ontario in FY2008-2009 identified 11.4 million adults. Nearly half of the cohort was female and 16.1% were aged 65 or older. (Table 1) Approximately 3.2 M (28%) did not use the healthcare system or had services with unclassified diagnoses (ACG = 5100); their average ACG weight was 0.17. Conversely, 45,383 adults (0.4%) were categorized into the highest average ACG weight of 12.61 (ACG = 5070). Figures 1A-1C comprise the provincial dashboard. Figure 1A displays the distribution of number of adults who are categorized in the various ACG categories. The second largest ACG category is adults older than 34 who are expected to have acute minor issue(s) (e.g. ear infections, sore throats, etc.), comprising 10.9% of the population (ACG = 4100). In the lower 4 resource utilization quintiles, the ACG weight is less than twice (2.00) the average adult.Figure 1B displays the total provincial health system costs in Canadian dollars for all adults within each of the ACG categories, totalling $15.8B of direct costs from hospital, fee-for-service physician and ancillary service billing, and ED services. Based on ACG weights, the sickest 1%, 5% and 15% of the adult population will use about 10%, 30% and 50% of total health system costs respectively. The largest 5 ACG categories with the most aggregate costs to the health system are: acute minor issues for adults >34 (ACG = 4100); 0–1 major ADG, age >34, and 6–9 other ADG combinations (ACG = 4910); 2 major ADG, age >34, and 6–9 other ADG combinations (ACG = 4920); 1 major ADG, age >44, and 4–5 other ADG combinations (ACG = 4420); and the 4+ major ADGs, age > 18, and 10+ other ADG combinations (ACG = 5070). Health system costs directly relate to both the number of adults within each group and the severity of the adults’ conditions. Hospital services comprise the largest percentage of cost within each ACG, followed by physician services. ED costs comprise a very small fraction of total costs within each ACG.Figure 1C displays the average cost per category (weight = 12.6) was $17,271. The average yearly cost per adult (ACG weight of 1) was $1,382. Similar to total system costs, hospital services comprise the largest percentage of costs.

**Table 1:** **Cohort demographics and ACG assignment by ACG weight**

		N	%	ACG weight	Resource utilization quintile
**Overall**					
		11,391,085	100	1	
**Sex**					
	Female	5,894,366	51.8	1.08	
	Male	5,496,719	48.3	0.92	
**Age**					
	*19-34*	3,221,862	28.3	0.56	
	*35-44*	2,408,590	21.1	0.7	
	*45-54*	2,303,950	20.2	0.95	
	*55-64*	1,626,276	14.3	1.26	
	*65-74*	979,727	8.6	1.72	
	*75-84*	623,531	5.5	2.3	
	*85+*	227,149	2	2.48	
**ACGs, sorted by lowest to highest illness severity weight**					
5100	No or Only Unclassified Diagnoses & Non-Users	3,189,204	28	0.17	0
1600	Preventive/Administrative	185,342	1.6	0.33	1
600	Likely To Recur, with Allergies	28,658	0.3	0.36	1
700	Asthma	20,604	0.2	0.37	1
300	Acute Minor, Age 6+	633,452	5.6	0.38	1
500	Likely To Recur, without Allergies	282,943	2.5	0.39	1
2200	Acute Minor: Age > 5,with Allergy	38,494	0.3	0.44	1
400	Acute: Major	407,466	3.6	0.49	1
2100	Acute Minor: Age > 5,w/out Allergy	289,867	2.5	0.49	2
3900	Acute Minor: Male, Age 18-34	106,375	0.9	0.51	2
1300	Psychosocial, without Psychosocial Unstable	124,842	1.1	0.53	2
1200	Chronic Specialty, Unstable	17,645	0.2	0.55	2
1000	Chronic Specialty	6,545	0.1	0.58	2
1800	Acute Minor and Acute Major	404,069	3.6	0.59	2
2800	Acute Major And likely To Recur	189,580	1.7	0.6	2
2400	Acute Minor and Eye/Dental	9,620	0.1	0.6	2
2500	Acute Minor, Psychosocial, Without Unstable	94,404	0.8	0.61	2
1100	Ophthalmological/Dental	17,678	0.2	0.62	2
3300	Acute Minor: Age > 12, with Allergies	44,122	0.4	0.67	2
4000	Acute Minor: Female, Age 18-34	108,935	1	0.73	2
900	Chronic Medical, Stable	311,434	2.7	0.76	2
3200	Acute Minor: Age > 12,w/out Allergies	327,428	2.9	0.76	3
3400	Acute Minor/Likely To Recur/Eye & Dental	6,164	0.1	0.77	3
4310	4-5 Other ADG Combos, Age 18–44, No Major ADGs	162,851	1.4	0.79	3
4710	6-9 Other ADG Combos, Male, Age 18–34, No Major ADGs	6,218	0.1	0.8	3
2300	Acute Minor and Chronic Medical: Stable	191,521	1.7	0.81	3
3500	Acute Minor/Likely To Recur/Psychosocial	79,984	0.7	0.84	3
4320	4-5 Other ADG Combos, Age 18–44, 1 Major ADG	125,136	1.1	1.07	3
4100	Acute Minor: Age >34	1,245,235	10.9	1.23	3
4810	6-9 Other ADG Combos, Female, Age 18–34, No Major ADGs	19,406	0.2	1.25	4
1730	Pregnancy: 2–3 ADGs, 1+ Major ADGs	8,041	0.1	1.26	4
3700	Acute Minor & Major/Likely to Recur/Psychosocial	139,182	1.2	1.29	4
4720	6-9 Other ADG Combos, Male, Age 18–34, 1 Major ADGs	11,186	0.1	1.3	4
4410	4-5 Other ADG Combos, Age >44, No Major ADGs	334,873	2.9	1.31	4
3600	Acute Minor/Maj/Likely to Recur/Chronic Med:Stable	236,710	2.1	1.35	4
800	Chronic Medical, Unstable	62,924	0.6	1.41	4
1750	Pregnancy: 4–5 ADGs, 1+ Major ADGs	21,750	0.2	1.49	4
4820	6-9 Other ADG Combos, Female, Age 18–34, 1 Major ADG	22,384	0.2	1.61	4
1710	Pregnancy: 0–1 ADGs	15,661	0.1	1.66	4
4330	4-5 Other ADG Combos, Age 18–44, 2+ Major ADGs	29,298	0.3	1.67	4
2600	Acute Minor: Unstable without Stable	10,486	0.1	1.69	4
1400	Psychosocial, with Unstable, without Stable	26,273	0.2	1.76	4
1720	Pregnancy: 2–3 ADGs, No Major ADGs	68,678	0.6	1.76	4
2700	Acute Minor: with Unstable & Stable	6,908	0.1	1.84	4
1500	Psychosocial, with Unstable and Stable	10,742	0.1	1.84	4
1740	Pregnancy: 4–5 ADGs, No Major ADGs	61,586	0.5	1.86	4
1770	Pregnancy: 6+ ADGs, 1+ Major ADGs	40,171	0.4	2.05	5
1760	Pregnancy: 6+ ADGs, No Major ADGs	34,666	0.3	2.13	5
4420	4-5 Other ADG Combos, Age >44, 1 Major ADGs	404,582	3.6	2.16	5
4910	6-9 Other ADG Combos, Age >34, 0–1 Major ADGs	507,762	4.5	2.3	5
4830	6-9 Other ADG Combos, Female, Age 18–34, 2+ Major ADGs	10,758	0.1	2.68	5
4730	6-9 Other ADG Combos, Male, Age 18–34 2+ Major ADGs	9,737	0.1	2.76	5
5040	10+ Other ADG Combos, Age 18+, 0–1 Major ADGs	33,410	0.3	3.11	5
4430	4-5 Other ADG Combos, Age >44, 2+ Major ADGs	136,748	1.2	3.29	5
4920	6-9 Other ADG Combos, Age >34, 2 Major ADGs	237,874	2.1	3.94	5
5050	10+ Other ADG Combos, Age 18+, 2 Major ADGs	43,637	0.4	5.08	5
4930	6-9 Other ADG Combos, Age >34, 3 Major ADGs	83,427	0.7	5.72	5
5060	10+ Other ADG Combos, Age 18+, 3 Major ADGs	42,381	0.4	7.45	5
4940	6-9 Other ADG Combos, Age >34, 4+ Major ADGs	18,533	0.2	7.9	5
5070	10+ Other ADG Combos, Age 18+, 4+ Major ADGs	45,383	0.4	12.61	5

## Discussion

Our study applied a case-mix and costing method to a large population-based sample to analyze the predicted health care needs, and then follow that population to calculate the actual utilization costs. This analysis was summarized as a health system dashboard of the sickest and highest cost users in the system, which acts as one measure of health system performance. Unsurprisingly, the sickest and most expensive adults had multiple major illnesses and multiple combinations of comorbidities. Similar to the US, a small percentage of our Ontarian study population (15%) use a disproportionate amount of the total health care system costs (48%).

### The dashboard can help to partially measure the Triple Aims

**Figure 2:**
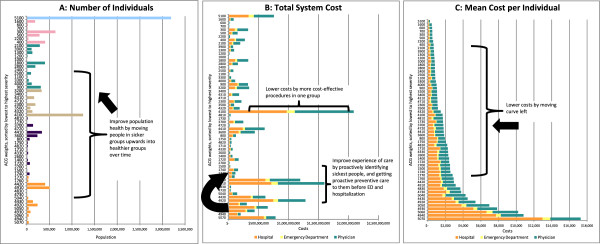
**Using the dashboard to help achieve the Triple Aim.**

We propose that the dashboard can measures 2 of the 3 dimensions of the Triple Aim, namely health system costs and population health. (Figure 2) First, the dashboard indicates lower system costs over time when the cost curve in Figure 2C shifts to the left (so average health costs/person are less) or when any ACG category in Figure 2B is shortened (so total system costs for an ACG category is reduced). Second, the dashboard indicates a healthier population over time when the healthcare utilization curve in Figure 2A shifts upwards (more people move upwards into ACG categories with less healthcare use) rather than downwards (more healthcare use). While past and predicted healthcare utilization is a proxy for population health, we recognize it does not truly capture all aspects of health, such as quality of life. Nonetheless, measurement of the Triple Aim, even partially, is important.

### The dashboard can help policymakers to achieve all of the Triple Aims

In addition to measurement, we also propose that the dashboard’s information can support health system planners to manage their health delivery systems more effectively. The dashboard represents a profile of the illness burden and healthcare needs of a population at a given time, and is thus a useful tool to predict the need for future healthcare services. By using this data, planners and providers can proactively intervene with appropriate policy, programs, and clinical and social interventions for the highest risk patients or the patients who are most likely to benefit so that ideally the same population is healthier the next time the dashboard is measured. Thus, the dashboard would need to be reported regularly (e.g. quarterly or biannually) in a continual feedback loop to indicate changes over time. Using the dashboard in this way would be supported by evidence that show the effectiveness health system measures depends on the extent that it reflects the goals of the health system, the quality of the data, and the incentives for providers to scrutinize and act on the data [48]. Below, we provide an example of how the dashboard could be used at various levels to support the achievement of the Triple Aim.

-  
**Reduce costs:** First, to achieve lower system costs and move the cost curve left, (See Figure 2C) policymakers and providers could use the dashboard to support cost-effective improvements in care (e.g. new technologies, diagnostic equipment, or surgical techniques), such as in ACGs with many individuals even if they are not the most complex cases. The dashboard might also identify medical provider specialties that ought to be high priority for better alignment with financial incentives that focus on patient outcomes or bundles of care, rather than number of visits, as in fee-for-service. Second, policymakers and providers can act to reduce costs within individual ACG categories. For highest-needs or highest-costs individuals, they could support interventions that provide patients with additional care support, intensive case management, or early referral to palliative care as appropriate, all of which can avoid unnecessary hospitalizations and ED visits. For medium- and low-risk individuals, they could support interventions that maintain or improve health, such as self-care, healthy nutrition, exercise, and wellness programs, which can reduce costs within an ACG category. (See Figure 2B)
-  
**Improve population health:** The dashboard can present data to providers at an individual or group practice level about their patient rosters to determine if their roster is getting healthier over time (See Figure 2A). Providers and policymakers could use the information to focus on particular health conditions, regions, or populations that could benefit from targeted interventions, and the size and scale required for such interventions. For the sickest population, they could refer them to interventions or supports that prevent the worsening of their condition (e.g. falls prevention), or help them manage complex conditions (e.g. intensive case management or nurse coaching). Just as importantly, for the medium and low-risk population, providers could encourage interventions that promote prevention and wellness, improve health, and reverse the disease progression, such as self management and exercise programs.
-  
**Improve patient care experience:** The dashboard does not measure care experience directly. However, the dashboard’s information potentially can help providers improve care experience and overall health. For instance, the dashboard could report to providers the high-risk, medically complex patients they are the most responsible physician for. The providers could then work proactively, rather than reactively, to intervene and develop multidisciplinary care plans for higher need individuals to prevent worsening of the condition(s) rather than wait for them to arrive in the hospital or their office with an issue. Thus the dashboard can support integration with the broader health system and health care team to improve the care experience for the patient.

The dashboard marries both an economic analysis with a case-mix adjustment analysis to help user predict future costs in relation to predicted illness burden and health care needs. When the dashboard is compared over multiple time periods, it can provide a dynamic tool to help measure population health and health system performance. A key strength of the dashboard is that it can be adapted to profile multiple populations: it could be presented at a macro level (e.g. NHS regional teams or state Medicaid insurance program) for high-level planning and resource allocation, but could also be useful at a micro level (e.g. small health region, community, or small group practice) where sample sizes are small enough for health care providers to assume responsibility for patients in an ACG. Moreover, the dashboard can be adapted to present data by age (e.g. children), by disease or condition (e.g. cancer or heart disease), by geography, income, etc. Thus disease specific organizations, or community programs that focus on particular ages or needs, can also use a similar methodology to target high risk patients, as well as track impact and improvement on the population’s health. The dashboard relies on case-mix methods, which have the advantage of typically relying on existing administrative data already available. Moreover, large administrative data can produce population-based profiles, connect across multiple sectors, and compare across regions to allow for a broader health system assessment. Finally, the dashboard is not constrained by the ACG case-mix method. Other case-mix methodologies could be applied in a similar way to population-based samples.

The dashboard could be useful to policymakers, health system planners, and providers for measuring health system performance, especially in countries with largely single payer health systems, such as in Canada, UK, or other European Union countries. Many federally and regionally funded programs could report this dashboard indicator publically and regularly in order to manage region-wide initiatives and to facilitate quality improvement. In the US, the dashboard would be especially useful in “closed” group model systems or capitated managed care organizations, where they provide comprehensive care team is within the same health delivery system, so care information such as medical records can be shared easily. It would also be useful in open group models, because the dashboard is not setting specific and can support providers to better coordinate and integrate care across settings and providers.

While the dashboard might be unique and useful, it needs further development in applicability and accountability. We did not explore the ACG categories by provider practice group, by region, by children versus adult, or by disease type, though have planned other subsequent research to do this. The costing methodology could be more comprehensive and inclusive, and does not include privately obtained services. Cost and utilization by ACG does not determine quality of care. Case-mix measures are dependent on observed morbidity and provider diagnostic coding, which may be biased by unobserved morbidity and coding behaviours [49]. Moreover, we have yet to explore the predictive nature of the dashboard for different outcomes from one year to the next. This paper showed a snapshot of one year, and did not explore the true dynamic nature of the dashboard over multiple time periods. Prior research has shown the ACGs alone are predictive of 40% of same year’s and 14% of next year’s total costs [42]. Other versions of the ACG system include prescription drugs when assigning categories, which might improve predictive ability, though this was not done in this study. Further research that combines ACG data with other administrative data, such as clinical, health behavior, drug data, or survey data about quality of care, may increase its predictive ability and should be explored.

The application of case-mix methodology to administrative, population based data is not novel. In the public domain, many research studies applied ACGs to various populations for the purposes of case-mix adjustment and reimbursement, practice variation, provider profiling, and the management of disease populations [33–37, 50]. However, these studies do not aim to indicate health system performance with respect to the Triple Aim. In the proprietary domain, many managed care organizations and health insurers in the US, responsible for healthcare delivery to large populations, have likely adapted various case-mix methods to predict future expenditures and to measure health system performance, at least in the populations they serve. However, to our knowledge, the results of such analyses, especially the costing data, have not been made publicly available due to proprietary nature of the data. We believe this dashboard is a unique contribution in that it applies a standard case-mix approach—commonly used for confidential reimbursement, insurance rate setting, and practitioner profiling—in a way that can be used by health system planners to measure health system performance and inform quality improvement activities, whether organizations choose to make the data public or not. To measure more dimensions of the Triple Aim and health system performance more broadly, the dashboard should be integrated and used in combination with other quality measures.

## Conclusion

In summary, the health system performance dashboard, developed via case-mix methods, can help policymakers, planners, and providers better manage their health delivery systems because it measures two of three of the Triple Aim goals at a system level. Moreover, when fed back appropriately to providers and policymakers, the health system performance dashboard has the potential to support appropriate resource allocation and related quality improvement in the system and ultimately help to achieve the Triple Aim.

## Abbreviations

ACG: Adjusted Clinical group
ADG: Ambulatory Diagnostic Grouper
ED: Emergency department
FY: fiscal year
UK: United Kingdom
US: United States.
